# Dietary Plant Saponins and Exercise-Induced Fatigue: Structural Characteristics, Bioavailability, Efficacy, Physiological Mechanisms, and Implications for Functional Foods

**DOI:** 10.3390/nu18213581

**Published:** 2026-10-29

**Authors:** Yien Xiang, Jun Wu, Jiacheng Wu

**Affiliations:** Jilin University Engineering Laboratory for Translational Medicine of Hepatobiliary and Pancreatic Diseases, Norman Bethune Health Science Center of Jilin University, Jilin University, Changchun 130021, China; xiangyien@jlu.edu.cn (Y.X.); wujun@jlu.edu.cn (J.W.)

**Keywords:** exercise-induced fatigue, dietary saponins, ginsenosides, gypenosides, structure–activity relationship, gut microbiota, mitochondrial function

## Abstract

**Background**: Exercise-induced fatigue involves a task-dependent reduction in the capacity to sustain physical performance, an increase in perceived fatigue during or after exercise, or both. Dietary plant saponins are amphiphilic glycosides widely distributed in edible plants and botanical preparations, but evidence regarding their effects on exercise-related fatigue remains heterogeneous across food chemistry, exercise physiology, and pharmacological research. **Scope and approach**: This narrative review critically evaluates dietary plant saponins using a composition–exposure–outcome framework. The framework links botanical source and processing history with structural and chemical definition, gastrointestinal transformation and systemic exposure, and clearly defined exercise-related outcomes. Human, animal, and mechanistic evidence are considered separately according to exercise-related outcome domain, and principal human trials are qualitatively appraised for randomization/blinding, sample size/attrition, outcome selection, and potential bias, with emphasis on distinguishing preparation-level effects from evidence attributable to individual saponins. **Key findings and conclusions**: Preclinical studies of saponin-rich preparations have reported improvements in exercise duration, glycogen-related indices, lactate-related measures, and changes in mitochondrial, inflammatory, or redox-associated markers. Human evidence remains limited and includes both preparation-level findings and emerging compound-specific evidence from purified ginsenoside Rg1; however, the available Rg1 trials are small, predominantly acute, and require independent replication. Preparation-level evidence is available for *Panax ginseng*, *Gynostemma pentaphyllum*, and *Panax notoginseng*, whereas controlled *Tribulus terrestris* trials have not demonstrated consistent ergogenic effects. Current evidence does not support a simple structure–activity relationship based solely on total-saponin content, sugar-chain number, aglycone class, or stereochemistry. Instead, processing-dependent composition, gastrointestinal deglycosylation, microbial transformation, and systemic exposure must be considered together when interpreting biological responses. The major unresolved question is therefore not whether saponins influence fatigue-related pathways, but whether chemically defined preparations achieve biologically relevant exposure levels that reproducibly improve prespecified exercise outcomes. Future studies should integrate compositional standardization, parent and metabolite exposure assessment, physiologically meaningful exercise endpoints, and food-format feasibility.

## 1. Introduction

Exercise-induced fatigue can involve a reduced capacity to generate force or sustain physical performance, an increase in perceived fatigue during or after exercise, or both. At the skeletal-muscle level, adenosine triphosphate (ATP) turnover, phosphocreatine availability, glycogen use, metabolite accumulation, ion handling, and excitation–contraction coupling interact to reduce force-generating capacity. At the organismal level, substrate and oxygen delivery, motor drive, thermal strain, and perceived fatigability shape exercise tolerance [1,2,3]. This multilevel nature also cautions against inferring a broad “anti-fatigue” effect from either a single biochemical surrogate or an isolated protocol-dependent performance endpoint. Blood lactate and redox biomarkers require context-specific interpretation [4,5], while animal fatigue models rely on protocol-specific definitions and multiple monitoring indicators [6]. Evidence for an anti-fatigue effect is more informative when a relevant performance or recovery phenotype is accompanied by mechanistic measurements that help explain, rather than substitute for, that phenotype.

Nutritional interventions can modify several determinants of exercise tolerance, including substrate availability, acid–base balance, neuromuscular function, and perceived effort [7]. Caffeine, creatine, and beta-alanine illustrate how sports-nutrition ingredients can progress from a mechanistic rationale to standardized dosing and repeated human performance studies [8,9,10]. Botanical extracts and multicomponent phytochemical preparations have also been investigated in this context, but their human performance evidence is generally more heterogeneous and product-specific [11]. Among these compounds, saponins have received attention because their hydrophobic aglycone and one or more hydrophilic sugar chains confer amphiphilic physicochemical properties and substantial structural diversity. These features can influence interactions with membranes, proteins, lipids, and food interfaces, although the nature and magnitude of these interactions are structure dependent [12,13]. Dietary saponins occur in grains, legumes, leafy plants, and several botanicals used as foods or traditional medicines [13,14]. Among the saponins investigated in exercise-related contexts, human evidence remains limited and is available mainly for selected standardized botanical preparations, with emerging evidence from purified individual saponins, whereas evidence for many other plant sources remains predominantly preclinical.

Existing reviews have summarized the pharmacological activities of plant saponins [14,15], fatigue-related evidence for *Panax ginseng* [16], and processing and food applications [15,17]. Interpretation of exercise studies nevertheless requires attention to three issues. Whole extracts, saponin-enriched fractions, and purified compounds differ in composition, and extracts also contain non-saponin constituents [18,19,20,21]. Oral exposure can vary with solubility, permeability, and intestinal or microbial transformation [14,22,23]. Finally, findings from non-exercise models do not directly establish an effect on exercise-induced fatigue.

**Figure 1:**
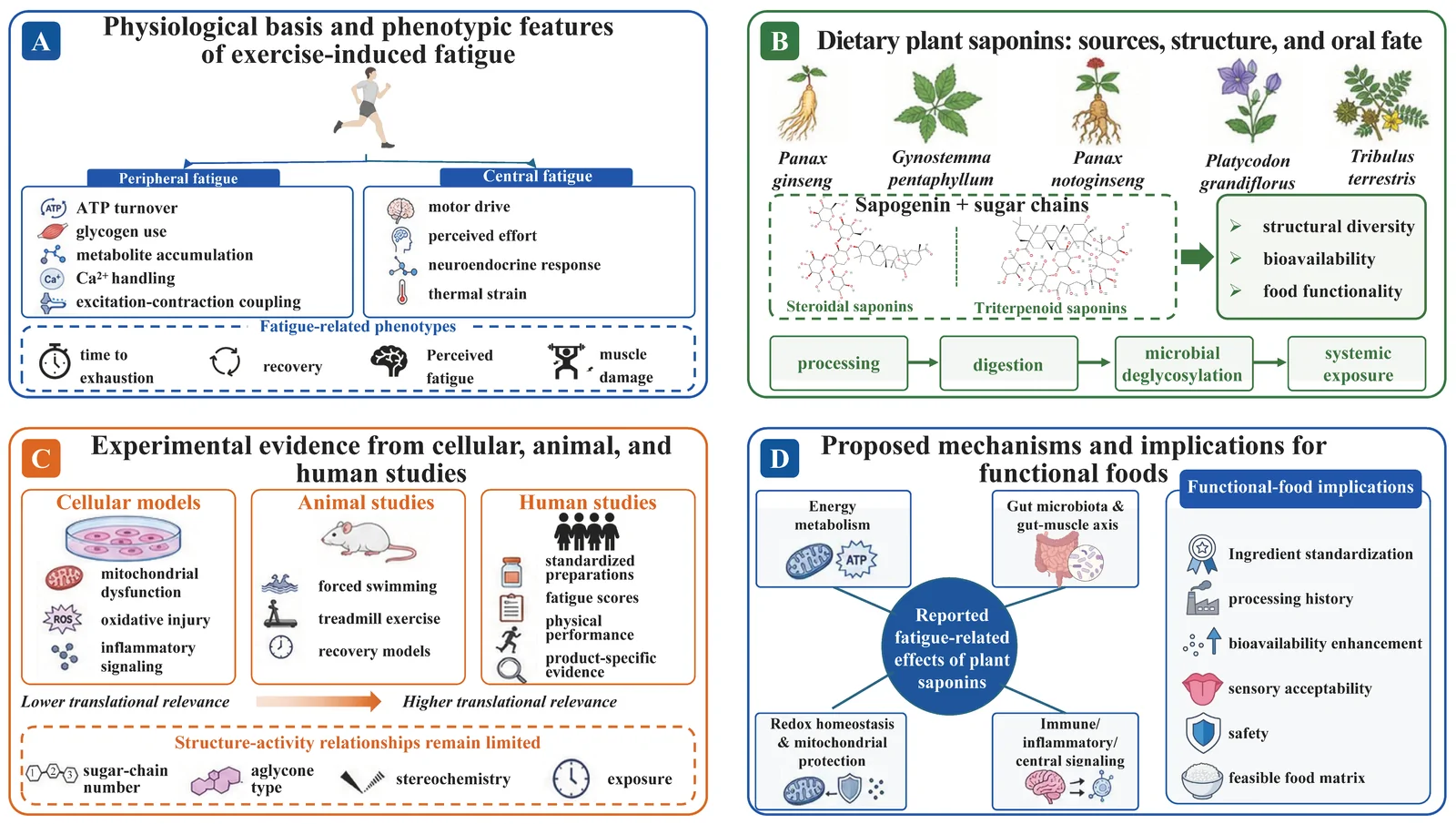
Overall framework of this review on dietary plant saponins and exercise-induced fatigue. (**A**) Physiological basis and phenotypic features of exercise-induced fatigue; (**B**) food-relevant plant sources, structural diversity, and oral fate of dietary saponins; (**C**) experimental evidence from cellular, animal, and human studies; (**D**) proposed physiological mechanisms and implications for functional-food development.

This review examines dietary plant saponins in relation to exercise-induced fatigue. The primary population of interest is healthy adults who undertake physical exercise, including untrained adults participating in exercise studies, recreationally active adults, and trained individuals. The outcomes considered are exercise performance, perceived fatigue, and post-exercise recovery. We discuss saponin composition, processing, gastrointestinal transformation, and systemic exposure to help interpret the reported effects. Human, animal, and cellular findings are considered separately, and effects of botanical preparations are distinguished from those attributable to individual saponins. Implications for functional foods and sports nutrition are assessed in relation to the populations, preparations, and conditions studied (Figure 1).

## 2. Literature Identification, Study Selection, Data Extraction, and Critical Appraisal

This narrative review evaluates dietary plant saponins in relation to exercise-induced fatigue. No pooled effect estimates were calculated. Relevant literature was identified through searches of PubMed, Web of Science Core Collection, and Scopus. The final database searches were conducted on 1 September 2026, covering records from database inception through that date. Search terms were selected to capture studies related to plant saponins, exercise-related fatigue, biological exposure, and functional-food applications. The search strategy combined terms related to saponins (including “saponin”, “ginsenoside”, “ginsenoside Rg1”, “gypenoside”, “notoginsenoside”, “platycodin”, and plant-derived saponins), exercise outcomes (including “exercise”, “physical performance”, “endurance”, “fatigue”, “anti-fatigue”, “exercise-induced fatigue”, and “recovery”), and translational aspects (including “bioavailability”, “pharmacokinetics”, “metabolism”, “biotransformation”, “gut microbiota”, and “functional food”). Additional relevant studies were identified through reference list screening and citation tracking of key publications.

Study eligibility was assessed by Yien Xiang, and the selected records were checked by Jun Wu. Data for studies retained in the detailed synthesis were extracted by Yien Xiang and checked against the original reports by Jun Wu. Extracted information included intervention identity and dose, chemical standardization, study design and comparator, participant or model characteristics, sample size and attrition, exercise or fatigue context, main outcomes, effect estimates and measures of uncertainty where reported, and relevant exposure or mechanistic variables. Any discrepancies were resolved through discussions among Yien Xiang, Jun Wu, and Jiacheng Wu. Human clinical trials, animal exercise models, and exercise-induced fatigue studies were prioritized when they directly evaluated performance, perceived fatigue, recovery, or exercise-associated physiological responses. Human findings are interpreted in relation to the health and training status reported in each study. Studies focusing on cellular systems or non-exercise fatigue models were included when they provided mechanistic information relevant to exercise physiology, including pathways associated with energy metabolism, mitochondrial regulation, redox homeostasis, inflammation, or central fatigue. However, these studies were interpreted as evidence for biological plausibility rather than direct evidence of exercise efficacy.

Plant sources were selected based on three considerations: (i) relevance to dietary or food-related applications; (ii) availability of exercise-related fatigue, performance, or recovery studies; (iii) representation of chemically distinct saponin classes. Accordingly, the review focused primarily on *Panax ginseng*, *Gynostemma pentaphyllum*, *Panax notoginseng*, *Platycodon grandiflorus*, and *Tribulus terrestris*, which collectively represent major triterpenoid and steroidal saponin categories and encompass different levels of human and preclinical evidence. Other food-relevant saponin sources lacking direct exercise evidence, such as Quillaja, were discussed only as food-functionality or formulation comparators and were not treated as evidence of exercise efficacy.

Because dietary saponin preparations vary substantially in chemical definition, studies involving whole botanical extracts, saponin-enriched fractions, and purified compounds were considered separately during interpretation. Particular attention was given to differences in botanical identity, processing history, structural composition, administration route, and exposure characteristics. Non-exercise fatigue models, including chronic fatigue unrelated to exercise, disease-related fatigue, or postoperative fatigue models, were not considered equivalent to exercise-induced fatigue and were discussed only when they provided mechanistic insights relevant to exercise physiology. Evidence interpretation therefore emphasized the distinction between preparation-level effects, individual saponin attribution, indirect mechanistic findings, and translational relevance.

Methodological quality was appraised qualitatively rather than converted into a numerical score, consistent with the narrative-review design. For the principal human trials, appraisal considered randomization, blinding, comparator selection, sample size, attrition and analysis set, outcome selection, whether reported effects represented within-group changes or between-treatment differences, reporting of effect estimates and uncertainty, chemical characterization of the intervention, and potential sources of bias, including funding or conflicts of interest. These considerations informed the strength of the narrative synthesis: greater interpretive weight was given to randomized, blinded, placebo-controlled studies reporting between-treatment effects on direct exercise-performance or clearly defined fatigue/recovery outcomes, whereas small studies, within-group-only findings, mechanistic endpoints without corresponding performance outcomes, incomplete attrition reporting, or poorly characterized preparations were interpreted more cautiously. No formal risk-of-bias score was assigned.

## 3. Exercise-Induced Fatigue: Definition and Physiological Basis

### 3.1. Definition, Assessment, and Exercise Context

#### 3.1.1. Definition and Outcome Assessment

In this review, exercise-induced fatigue refers to an exercise-related reduction in the capacity to generate force or sustain physical output, an increase in perceived fatigue during or after exercise, or both [2,24]. Performance fatigability is quantified as a decline in force, power, or another objective performance measure during or after a defined task, whereas perceived fatigue is assessed in humans using self-report measures [2]. Ratings of perceived exertion describe perceived exercise effort and are reported separately from fatigue ratings [24]. Central contributions involve reduced voluntary activation, whereas peripheral contributions involve impaired force generation through processes at or distal to the neuromuscular junction [2,3].

Time to exhaustion, time-trial performance, swimming duration, and running distance are interpreted as task-specific performance outcomes. Animal performance tests also depend on workload, familiarization, motivational stimuli, and the criteria used to define exhaustion [6,25]. Maximal oxygen uptake (VO_2_max) is considered a measure of aerobic capacity, while post-exercise force recovery, soreness, and biochemical markers provide different information about recovery. Changes in lactate, creatine kinase, or redox markers alone do not establish reduced fatigue [4,5,26].

#### 3.1.2. Single Exercise Bouts and Repeated Training

Fatigue associated with a single exercise bout can be assessed during that bout or the subsequent recovery period. Assessment should specify the exercise protocol, the time of measurement, and whether the outcome is performance, perceived fatigue, or recovery of a measured function [2,26]. Fatigue during repeated training concerns fatigue that persists or accumulates across sessions, particularly when recovery between sessions is insufficient. Its assessment requires information on training load, recovery intervals, and changes in performance or perceived fatigue across sessions. Persistent fatigue alone should not be interpreted as overtraining syndrome [26].

For saponin interventions, supplementation duration and exercise context are considered separately. Several weeks of supplementation followed by an exercise test do not, by themselves, provide evidence on accumulated training fatigue. Studies of fatigue unrelated to exercise are treated as indirect evidence, and experimental models are classified by their protocols rather than by the use of the term “chronic fatigue” in their titles.

### 3.2. Physiological Mechanisms Involved in Exercise-Induced Fatigue

Skeletal muscle must continuously resynthesize ATP despite the small size of its intracellular ATP store. The relative contributions of phosphagen metabolism, glycolysis, and oxidative phosphorylation vary with exercise intensity and duration, while carbohydrate and lipid utilization depend on training status, nutritional state, oxygen delivery, and hormonal regulation [1,27]. Exercise fatigue can therefore reflect disturbances in metabolic homeostasis rather than depletion of a single fuel. Reduced glycogen availability can constrain carbohydrate flux during prolonged exercise, whereas accumulation of inorganic phosphate and perturbation of Ca^2+^ handling can impair contractile function, particularly during high-intensity exercises [1,4,27,28]. These processes provide physiological context for interpreting muscle and liver glycogen, ATP-related metabolites, and metabolic-enzyme measurements. Blood lactate, however, should not be treated as a direct index of contractile fatigue, because intracellular lactate accumulation per se is not a major cause of muscle fatigue [4].

Redox biology further illustrates why interpretation based on a single biomarker is inadequate. Reactive oxygen species (ROS) and reactive nitrogen species generated during exercise are not uniformly detrimental. Physiological oxidant signals participate in contraction, vascular responses, and training adaptation, whereas excessive or poorly controlled redox stress can damage proteins, lipids, and mitochondria and may contribute to impaired contractile function [29,30]. Human skeletal muscle activates Nrf2/Keap1-related responses during exhaustive exercise, which is consistent with dynamic regulation of redox signaling rather than a simple state of oxidative “overload” [31]. Accordingly, antioxidant-related effects are more appropriately evaluated in terms of redox homeostasis and functional outcomes than by ROS suppression or a single redox biomarker alone [5].

Mitochondrial quality control links oxidative energy production with redox regulation. Exercise-responsive mitochondrial biogenesis, dynamics, and mitophagy contribute to the remodeling and maintenance of skeletal-muscle mitochondria [32]. Mitophagy has been proposed as a potential target in exercise-induced fatigue, although direct human evidence linking mitophagy to exercise-related fatigue outcomes remains limited [33]. Skeletal muscle also communicates with other organs through myokines and metabolite signals, while the gut can influence energy availability, barrier integrity, inflammatory tone, and microbial-metabolite production [34,35]. These interactions provide a rationale for considering gut and immune pathways, but association with an exercise phenotype does not by itself establish mediation.

Central contributions to performance fatigability are task-dependent rather than reducible to a single neurotransmitter deficit [2,3]. Brain monoamines, afferent feedback from working muscle, thermal and metabolic signals, and perceived effort can influence voluntary drive, although their relative contributions vary with the task. Exercise also alters peripheral tryptophan–kynurenine metabolism, providing one route of communication among skeletal muscle, immune status, and the brain [36]. In saponin studies, changes in serotonin, dopamine, or related metabolites are therefore more informative when paired with measurements of motor drive, behavior, performance, or perceived effort. Neurotransmitter changes alone do not establish attenuation of central fatigue.

## 4. Dietary Saponins from Edible and Medicinal Plants: Sources, Processing, Structure, Physicochemical Properties, and Oral Fate

### 4.1. Sources and Structural Classification

Saponins are glycosides in which one or more sugar chains are attached to a lipophilic sapogenin. The major food-relevant classes are triterpenoid and steroidal saponins. Triterpenoid saponins include dammarane, oleanane, and related skeletons, whereas steroidal saponins are commonly based on spirostanol or furostanol aglycones. For the present topic, this chemical classification is more informative than a simple division between “medicinal” and “edible” plants because aglycone skeleton, glycosylation position, sugar composition, and stereochemistry can influence physicochemical behavior and metabolic fate [13,19].

*Panax ginseng* is a prominent saponin source in the fatigue literature reviewed here. Ginsenosides include protopanaxadiol (PPD)- and protopanaxatriol (PPT)-type dammarane saponins, together with less abundant oleanane and other structural types. Chemical surveys have catalogued hundreds of ginsenosides across P. ginseng tissues and steam-processed products, illustrating substantially greater structural diversity than what can be represented by total-saponin content alone [18,20]. “Ginseng saponins” should therefore not be treated as a chemically uniform intervention. Root, stem/leaf, berry, and fresh, white, red, or black ginseng can differ markedly in their saponin profiles [17,18,20]; similar total-saponin values therefore do not establish chemical equivalence. A recent review focused on the medicinal and edible value of *Panax ginseng* likewise emphasizes the relevance of constituent composition, processing, and product form [17].

**Figure 2:**
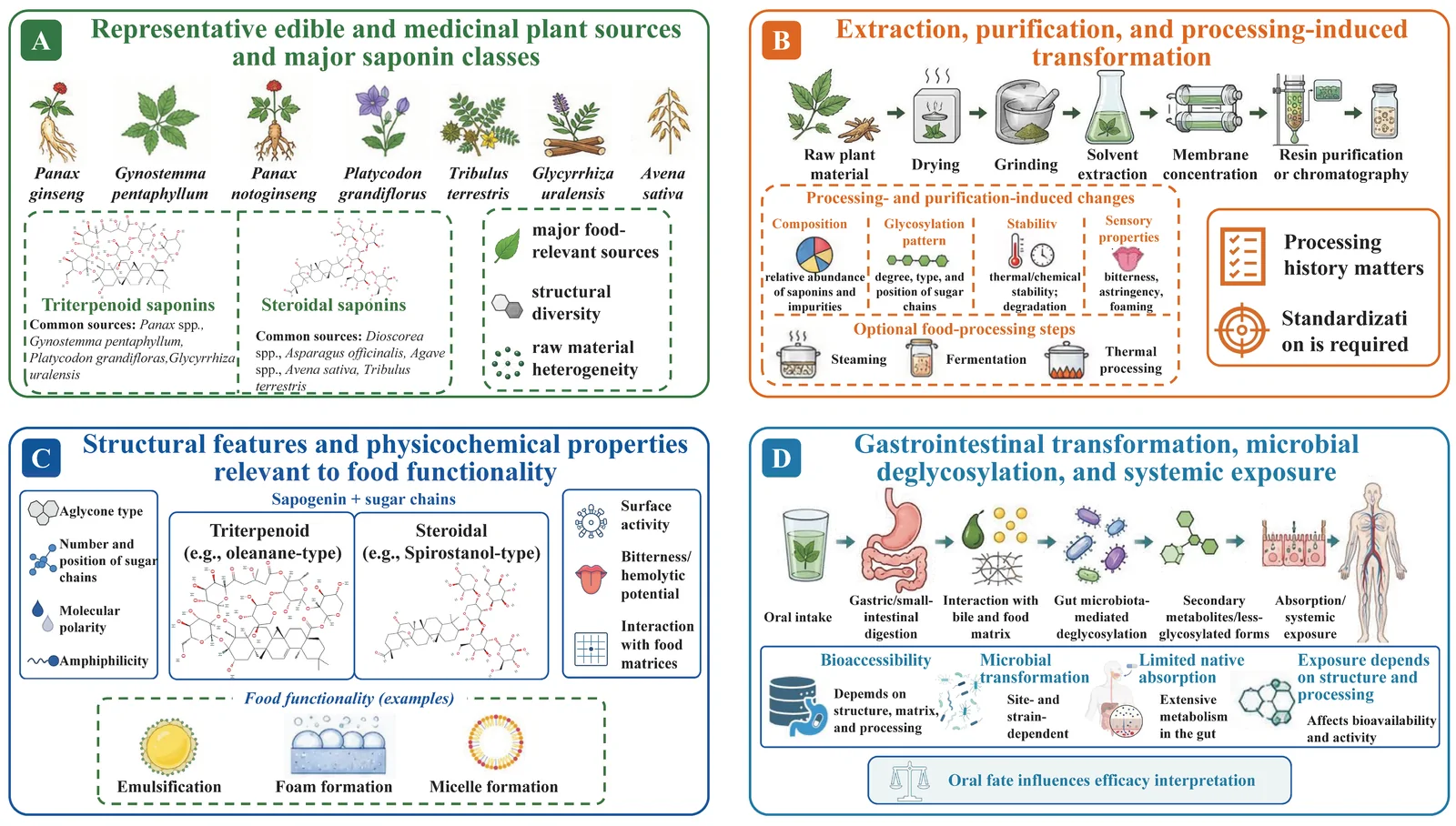
Food-relevant sources, processing, structural properties, and oral fate of dietary plant saponins. (**A**) Representative edible and medicinal plant sources and major saponin classes; (**B**) extraction, purification, and processing-induced transformation of plant saponins; (**C**) structural features and physicochemical properties relevant to food functionality; (**D**) gastrointestinal transformation, microbial deglycosylation, and systemic exposure.

*Gynostemma pentaphyllum* is another relevant plant used both as food and as traditional herbal material. Its gypenosides are predominantly dammarane-type triterpenoid saponins and show partial structural overlap with ginsenosides, while also containing numerous plant-specific glycosides [37]. Recent chemical profiling has revealed marked variation among *Gynostemma* species and geographic origins, reinforcing the need for compositional fingerprints rather than generic extract labels [38,39]. *Panax notoginseng* contains abundant dammarane saponins and stereoisomeric forms that become particularly relevant after processing [19]. *Platycodon grandiflorus*, which is used as both a food and traditional medicinal material, is rich in oleanane-type triterpenoid saponins such as platycodins [40]. *Tribulus terrestris* provides a contrasting structural class dominated by steroidal saponins and has also been investigated in exercise models [41,42]. Representative food-relevant plant sources and the major structural classes of their saponins are illustrated in Figure 2A.

Quillaja saponins are used as interfacial agents in foods, but this use does not establish an effect on exercise-induced fatigue [12,13]. For the saponin-containing preparations considered here, food applications also depend on sensory acceptability, physicochemical stability, feasible dose, compositional reproducibility, and regulatory status [13,23]. Representative sources and the main limitations of the current evidence are summarized in Table 1.

### 4.2. Extraction, Separation, Purification and Processing

Extraction and purification conditions affect the saponin composition of a preparation. A higher total extraction yield does not show that two preparations contain the same saponins in the same proportions [15,55]. Exercise studies should therefore report the preparation method and chemical profile to support comparisons among interventions.

Processing is especially relevant to both ginseng and *Gynostemma*. In ginseng, thermal treatment can transform highly glycosylated parent compounds into less polar saponins [22,43], whereas heat processing has also been shown to alter individual gypenoside profiles in *G. pentaphyllum* [47]. Fermentation or enzyme treatment can selectively remove sugar moieties, and such biotransformations are widely used to generate rare ginsenosides and other derivatives that occur at low abundance in the raw plant [22]. These compositional changes can alter oral fate, whereas their consequences for bitterness, solubility, and interfacial behavior are structure- and matrix-dependent and should be measured rather than assumed [13,22]. Consequently, comparisons among raw extracts, red ginseng, fermented ginseng, or enzymatically transformed saponins should not be interpreted as simple dose comparisons unless their chemical profiles are measured. The main extraction and purification steps and the compositional changes that may occur during subsequent processing are summarized in Figure 2B.

Analytical standardization is essential for reproducibility. Total-saponin colorimetric assays can be useful for process monitoring but do not establish molecular identity or chemical equivalence [15]. Liquid chromatography–tandem mass spectrometry (LC–MS/MS) and high-resolution mass spectrometry approaches can characterize multiple saponins and distinguish compositional variation associated with species or geographic origin [38,39]. Targeted LC–MS/MS approaches can also quantify processing-induced changes in individual saponin profiles [47]. Consistent with best-practice principles for botanical-extract characterization, studies of functional-food ingredients intended for exercise applications should, where feasible, report authenticated botanical identity, plant part and origin, extraction and processing history, extract yield, quantitative marker saponins, an appropriate chemical fingerprint, relevant non-saponin constituents, batch information, and storage stability [62]. Without this information, comparison and replication across efficacy studies remain difficult.

Purity should be interpreted in relation to the intended product. Extensive chromatographic purification is appropriate when causal attribution to an individual saponin is the experimental goal, but the resulting material no longer represents the original multicomponent food-grade matrix. Conversely, minimally refined extracts retain greater product relevance but increase challenges in batch reproducibility and attribution of biological effects [62]. For food-oriented research, a reasonable sequence is to define a reproducible food-grade extract and its chromatographic fingerprint, use fractionation or purified standards to examine which saponins are associated with the observed response, and then determine whether the selected chemical markers remain informative across batches of the food-grade ingredient. This links analytical characterization with reproducibility without implying that higher purity is necessarily preferable for a food product.

### 4.3. Structure, Physicochemical Behavior and Oral Fate

The amphiphilic structure of saponins contributes to their behavior in foods and biological systems (Figure 2C). The hydrophobic aglycone can associate with lipid domains, while sugar groups influence hydration, interfacial adsorption, and interactions with sterols or proteins [12,13]. High molecular mass and hydrogen-bonding capacity can limit membrane permeability, while low solubility or instability may further constrain oral absorption for some saponins [13,23]. Some Panax saponins occur as 20(S) and 20(R) epimers with different pharmacokinetic properties [19].

After oral intake, parent saponins may be deglycosylated by intestinal enzymes or the microbiota, producing metabolites with different permeability and biological activities [14,22]. The compounds reaching systemic circulation may therefore differ from those in the ingested preparation (Figure 2D). Human ex vivo data show variation in microbiota-mediated saponin biotransformation among 50 healthy donors, particularly for ginsenoside Rb1 [63]. Whether this variation contributes to differences in exercise responses remains unknown.

Food formulation can alter saponin stability, dispersion, release, and bioaccessibility [23]. Saponins may also cause bitterness or foaming [12,13]. Although food matrices affect digestion and bioaccessibility generally [64], direct pharmacokinetic comparisons of purified saponins and matched food preparations remain scarce. Delivery systems should therefore be evaluated for the specific saponin and intended dose using stability, release, bioaccessibility, and measured exposure to parent compounds and metabolites [23].

## 5. Experimental Evidence on Plant Saponins and Exercise-Related Fatigue

### 5.1. Cellular Models Relevant to Fatigue

No in vitro model fully reproduces exercise-induced fatigue, because isolated cells cannot capture whole-body exercise performance or perceived fatigue [2,24]. Cell systems can nevertheless reproduce selected processes relevant to exercise physiology, including mitochondrial stress, redox imbalance, inflammatory signaling, and substrate-handling responses. Saponins have been reported to influence several of these processes across cellular and non-exercise experimental systems, but such findings primarily support biological plausibility rather than direct exercise efficacy [14]. Accordingly, cellular models based on H_2_O_2_, lipopolysaccharide (LPS), hypoxia, or other stressors should be interpreted as mechanistic screening systems rather than as direct models of exercise-induced fatigue.

**Figure 3:**
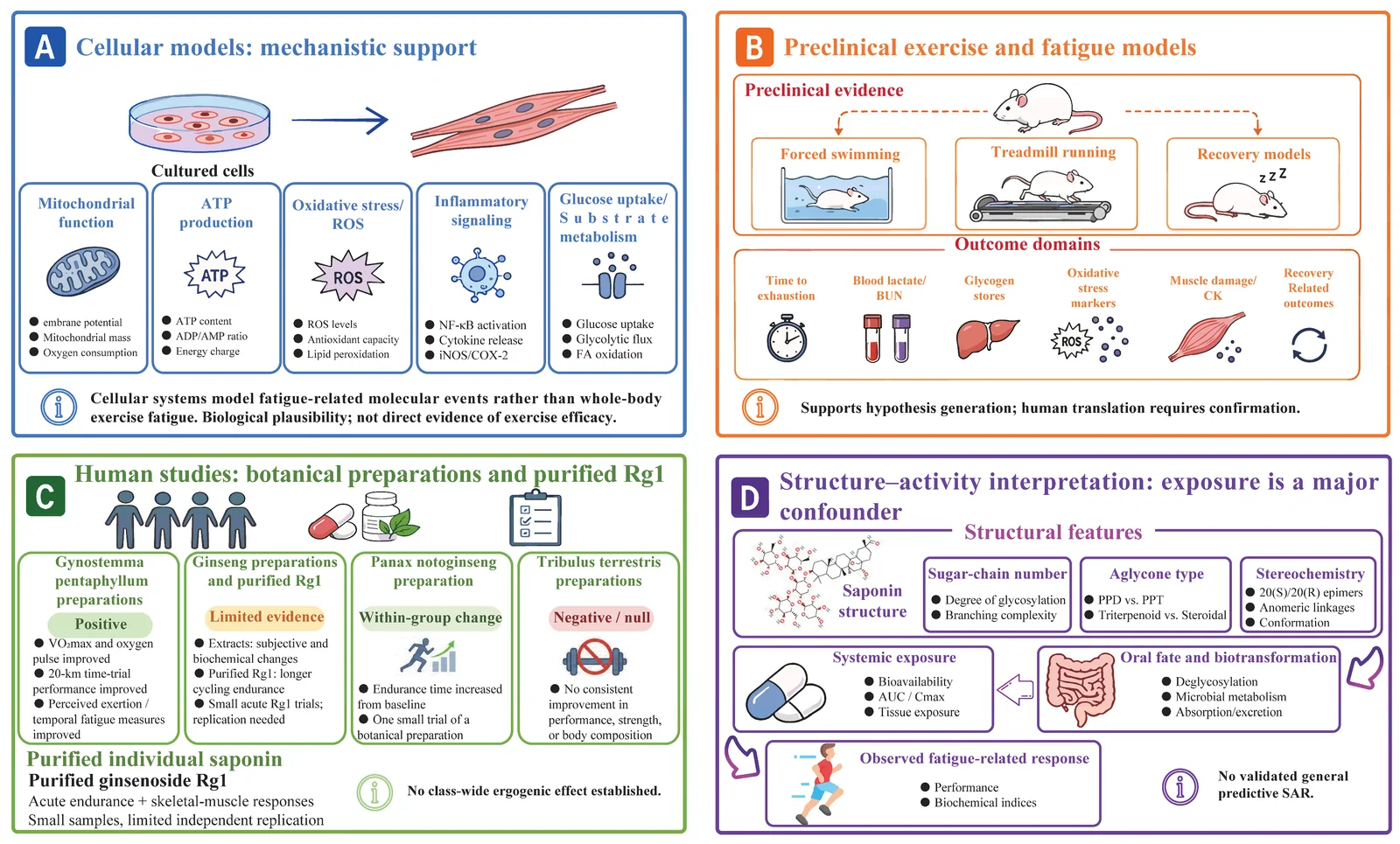
Evidence from cellular, animal, and human studies of plant saponins. (**A**) Cellular responses that support mechanistic hypotheses; (**B**) animal exercise models and measured outcomes; (**C**) human findings for botanical preparations and purified ginsenoside Rg1; (**D**) structural features and differences in oral transformation and exposure that limit structure–activity comparisons. Cellular responses and biomarker changes alone do not establish exercise efficacy, and the small acute Rg1 trials require independent replication.

Cellular studies could be made more relevant to exercise physiology by prioritizing contraction-based systems, such as electrically stimulated myotubes, and combining them with physiologically defined nutrient or stress conditions and measured exposure to parent saponins and gut-derived metabolites [65]. Relevant readouts include mitochondrial respiration, ATP turnover, glucose and fatty-acid flux, Ca^2+^ handling, redox state, and recovery after contraction. Such models can prioritize compounds and test pathway responsiveness, but should remain explicitly separated from organism-level evidence of fatigue or exercise performance. The main cellular models and their principal readouts in fatigue-related research are summarized in Figure 3A.

### 5.2. Human and Preclinical Evidence

#### 5.2.1. Human Evidence and Translational Relevance

Human studies have tested both saponin-containing preparations and purified ginsenoside Rg1, using different exercise protocols and outcomes.

The most extensively characterized human evidence for exercise-related outcomes comes from *Gynostemma pentaphyllum* preparations. A randomized, double-blind, placebo-controlled trial of a gypenoside L-containing *G. pentaphyllum* extract reported improvements in VO_2_max, oxygen pulse, perceived exertion, and temporal fatigue-related measures after 12 weeks of supplementation [51]. Another randomized crossover study in untrained young men reported faster 20 km cycling time-trial completion after four weeks of *G. pentaphyllum* supplementation, whereas time to exhaustion did not differ significantly from placebo [52]. These studies provide the strongest current human evidence linking a saponin-containing preparation with exercise performance-related outcomes. However, both interventions involved complex botanical preparations, and the specific contribution of individual gypenosides or other constituents to the observed effects remains unresolved.

Human trials of Korean red ginseng differ in whether fatigue was assessed after exercise. An eight-week placebo-controlled trial using graded cycling reported lower post-exercise subjective scores and lower lactate and creatine kinase levels with supplementation [45]. These outcomes did not establish improved exercise performance. Two other trials evaluated fatigue in daily life or in adults selected using traditional Chinese medicine deficiency criteria, without an exercise challenge [66,67]. These studies provide background on perceived fatigue but do not directly demonstrate an effect on exercise-induced fatigue. Differences in preparations, populations, and outcomes limit comparisons among these trials.

In contrast to botanical preparations, purified ginsenoside Rg1 provides a more direct opportunity to evaluate compound-specific effects in humans. In a randomized, double-blind, placebo-controlled crossover study, Hou et al. [68] administered 5 mg Rg1 on the night before exercise and a second 5 mg dose 1 h before exercise. In the performance cohort (*n* = 12), cycling time to exhaustion at 80% VO_2_max was greater with Rg1 than with placebo (38.3 ± 6.7 vs. 31.8 ± 5.0 min, *p* = 0.04), whereas VO_2_max was unchanged. The intervention was also associated with altered post-exercise glycogen recovery, lipid-peroxidation indices, citrate synthase activity, and inflammatory responses in skeletal muscle [68].

Wu et al. [69] used a randomized, double-blind, placebo-controlled crossover design with a single 5 mg dose of Rg1 administered 1 h before exercise. In a separate performance cohort (*n* = 12), Rg1 increased cycling time to exhaustion by 12% (1364 ± 145 vs. 1219 ± 135 s, *p* < 0.05) and total work by 13% (225 ± 33 vs. 199 ± 31 kJ, *p* < 0.05). A separate biopsy cohort (*n* = 12) showed reductions in senescence-associated β-galactosidase signals and changes in apoptosis-, macrophage-, and inflammatory-related responses after exercise [69].

These findings provide preliminary compound-specific human evidence for acute Rg1 effects, but their interpretation remains constrained by small sample sizes, acute dosing protocols, young male populations, limited independent replication, and the predominance of mechanistic endpoints. Importantly, the performance and biopsy experiments in Wu et al. were conducted in separate participant cohorts, so the observed cellular responses cannot be interpreted as established mediators of the endurance effect. Industry-linked funding was reported in both studies, and Wu et al. additionally disclosed a patent-related conflict of interest [68,69]. Accordingly, the current Rg1 literature supports emerging compound-specific evidence rather than an established or broadly generalizable ergogenic effect.

Beyond ginseng-derived evidence, evidence from other plant sources remains more limited but is important for balanced interpretation. In a randomized placebo-controlled study in untrained adults, Liang et al. reported an increase in cycling time to exhaustion from baseline after 30 days of *Panax notoginseng* supplementation [54]. The within-group change does not establish an advantage over placebo, and the small sample limits generalization. Conversely, human studies of *Tribulus terrestris* do not consistently support the positive findings reported in some animal models. Controlled human studies did not demonstrate a significant ergogenic advantage of Tribulus supplementation over placebo. Antonio et al. reported no benefit for body composition or resistance-exercise performance in resistance-trained males [60], while Ataei et al. reported no improvement in aerobic performance, muscle strength, or body composition following a detraining period [61]. These findings highlight the importance of considering negative human evidence when evaluating the translational relevance of botanical interventions.

Across the principal human trials, randomization and placebo control were common, and several studies used double-blind designs. However, sample sizes were generally modest, reporting of attrition and uncertainty varied, and some studies emphasized subjective, biochemical, or mechanistic outcomes rather than direct exercise-performance endpoints. In addition, several interventions were incompletely characterized chemically, and systemic saponin exposure was rarely measured.

Women are underrepresented in several trials that measured exercise performance. The Rg1 trials and the *G. pentaphyllum* crossover trial enrolled young men [52,68,69], while the larger *G. pentaphyllum* trial included 88 men and 12 women [51]. The Korean red ginseng cycling trial included more women than men and had mean participant ages above 50 years, but its findings concerned post-exercise subjective and biochemical outcomes [45]. Evidence remains insufficient to determine whether exercise-performance effects differ by sex, age, or training status. Findings from untrained or recreationally active participants cannot be assumed to apply to elite athletes.

Taken together, these trials do not establish an ergogenic effect shared by all dietary saponins. Differences among the human findings are summarized in Figure 3C.

#### 5.2.2. Preclinical Evidence from Exercise and Fatigue Models

Preclinical models represent the largest body of direct exercise-related evidence for dietary plant saponins, but their interpretation requires careful consideration of model relevance, dose feasibility, preparation complexity, and exposure differences between experimental animals and humans. These studies are valuable for identifying potential effects on exercise capacity, substrate metabolism, redox regulation, mitochondrial function, and recovery-related processes; however, they should primarily be interpreted as evidence supporting biological plausibility rather than direct confirmation of efficacy in human functional-food applications.

In mice, *Panax notoginseng* total saponins prolonged exhaustive-swimming time and altered blood lactate and liver and muscle glycogen [53]. Whole-ginseng preparations also improved exercise-related outcomes in mice [46] and in rats subjected to repeated loaded swimming [70]. A red-ginseng extract increased loaded-swimming and rotarod endurance and altered mitochondrial indices in mice exposed to cold-water swimming, rotarod exercise, and sleep deprivation [71]. The latter study therefore reflects combined stressors, which limits its application to fatigue caused by exercise alone. These findings concern the tested preparations and do not establish the contribution of individual saponins.

*Gynostemma pentaphyllum* preparations have also been investigated in several preclinical exercise and fatigue models. Studies using *Gynostemma* extracts or gypenoside-related preparations have reported improvements in treadmill or swimming performance accompanied by changes in mitochondrial function, oxidative status, inflammatory markers, or neurotransmitter-related responses [50,72]. A comparison between *Gynostemma* extract and gypenoside L suggested that both interventions could improve treadmill performance in mice, providing an informative comparison between a complex botanical matrix and a chemically defined component [50]. However, the exposure equivalence between the extract and isolated gypenoside L was not established, limiting interpretation of whether the observed effects were attributable to a specific saponin component.

Evidence from structurally distinct saponin classes remains comparatively limited. *Tribulus terrestris* saponin-rich preparations improved exercise performance in an overtraining rat model and were associated with endocrine and muscle-related changes [42]. *Platycodon grandiflorus*-derived saponins have been studied in both muscle-damage recovery and performance-related models. One study reported attenuation of eccentric exercise-induced muscle damage and inflammatory responses [57], whereas another observed improvements in grip strength, wheel-running activity, and time to exhaustion in mice [58]. These findings broaden the range of plant sources investigated beyond dammarane-type saponins, but remain limited by the absence of human replication and incomplete exposure–response characterization.

Interpretation of the animal findings is limited by multicomponent extracts, high experimental doses, incomplete chemical characterization, and sparse exposure measurements. The principal models and outcome domains are summarized in Figure 3B, while representative human and preclinical studies are compared in Table 2.

### 5.3. Structure–Activity Relationships: What Is Supported and What Remains Speculative

The available studies do not establish a quantitative structure–activity relationship for exercise-induced fatigue (Figure 3D). In a human pharmacokinetic study, bioconverted red ginseng produced greater systemic exposure to Rg3, Rk1/Rg5, F2, and compound K than conventional red ginseng [75]. This supports an effect of biotransformation on exposure, but does not show that fewer sugar residues improve exercise outcomes.

Aglycone comparisons provide preliminary evidence but remain insufficient for a general rule. In a direct PPD–PPT comparison included in this review, PPT significantly prolonged weight-loaded swimming time whereas PPD did not, although both altered fatigue-associated biochemical indices [73]. This discordance cautions against treating biomarker changes as equivalent to performance efficacy and is insufficient to establish a general PPD/PPT activity hierarchy. Steroidal saponins from *Tribulus terrestris* have also shown activity in an overtraining model [42], suggesting that reported fatigue-related effects are not limited to dammarane saponins. A major missing comparison is a common experimental platform in which structurally distinct saponins are tested at comparable systemic exposure.

Separate studies of 20(S)- and 20(R)-Rg3 reported fatigue-related effects, but differences in administration route, dose, and model prevent direct comparison of their effects [74,76]. These studies therefore do not establish which configuration is more effective for exercise-induced fatigue.

A major evidence gap is the lack of directly comparable experiments. Most published comparisons differ simultaneously in plant source, extraction method, dose, administration route, exercise protocol, and endpoint. Under these conditions, apparent structure–activity relationships may reflect pharmacokinetics or study design rather than intrinsic structural effects. A comparative platform could select a chemically coherent series, such as a parent ginsenoside, sequentially deglycosylated products, and 20(S)/20(R) epimers; quantify oral bioavailability; and test all compounds in the same standardized exercise model. The primary analysis could relate area under the concentration–time curve or tissue exposure to performance, with structural descriptors used to explain exposure differences. Such a design would help distinguish intrinsic structural effects from differences in exposure.

## 6. Physiological Mechanisms by Which Plant Saponins May Modulate Exercise-Induced Fatigue

### 6.1. Regulation of Energy Metabolism

The glycogen and lactate changes reported with saponin-containing preparations [46,53,70] have several possible explanations. Greater post-exercise glycogen can reflect higher pre-exercise stores, lower carbohydrate use at a matched workload, altered substrate selection, or differences in exercise output. Lower blood lactate can reflect changes in production, oxidation, clearance, or sampling time [4,27]. These possibilities should be distinguished before a specific metabolic mechanism is proposed.

AMP-activated protein kinase (AMPK) links cellular energy status with glucose uptake, fatty-acid oxidation, and mitochondrial adaptation [27]. In the *G. pentaphyllum* trial described in Section 5.2.1, oxygen flux in resting muscle samples was higher after extract supplementation than after placebo. During extract supplementation, AMPK Thr172 phosphorylation increased after 60 min of exercise relative to pre-exercise values [52]. In rats subjected to repeated loaded swimming, P. ginseng root extract altered glycogen availability and PI3K/Akt/mTOR-related proteins [70]. Neither study established that these pathways mediated the performance effects or that individual saponins accounted for the responses. Sampling during exercise, early recovery, and repeated training would help distinguish transient signaling responses from longer-term adaptation.

More informative food-ingredient studies would link metabolic outcomes to measured chemical exposure. Stable-isotope tracers, indirect calorimetry, muscle metabolomics, and high-resolution respirometry could determine whether a standardized saponin ingredient alters carbohydrate oxidation, lipid oxidation, lactate turnover, or mitochondrial coupling. These measurements would connect molecular signaling with exercise physiology more directly than additional untargeted panels of serum enzymes.

Mechanistic interpretation should also be phenotype-specific. In prolonged endurance exercise, carbohydrate availability and mitochondrial ATP production may be particularly relevant; in repeated high-intensity work, phosphocreatine recovery, buffering, and glycolytic capacity become more important; after eccentric muscle-damaging exercise, tissue repair and remodeling become increasingly relevant [4,27,77]. A saponin ingredient may therefore affect one fatigue phenotype while having little effect on another. Human trials should specify the intended use case, such as acute endurance, repeated-sprint performance, resistance-exercise recovery, or chronic training tolerance, before selecting mechanistic endpoints.

### 6.2. Gut Microbiota and Exercise-Related Responses

Microbial conversion of saponins affects exposure, as described in Section 4.3 and summarized in Figure 2D [14,22]. This section considers whether changes in the microbiota or intestinal function contribute to exercise performance or recovery.

Exercise-specific evidence remains limited but is available. Zhou et al. reported that a ginseng water extract in an exercise-induced fatigue rat model improved fatigue-associated biochemical and metabolic abnormalities together with gut-microbiota dysbiosis [78]. A recent synthesis of the broader exercise literature implicates microbial metabolites, intestinal permeability, inflammatory tone, and energy harvest in fatigue-related physiology [35]. Nevertheless, an association between microbiota composition and performance does not establish mediation. A taxonomic change after treatment may result from altered food intake, exercise tolerance, or host metabolism rather than being the cause of improved performance.

**Figure 4:**
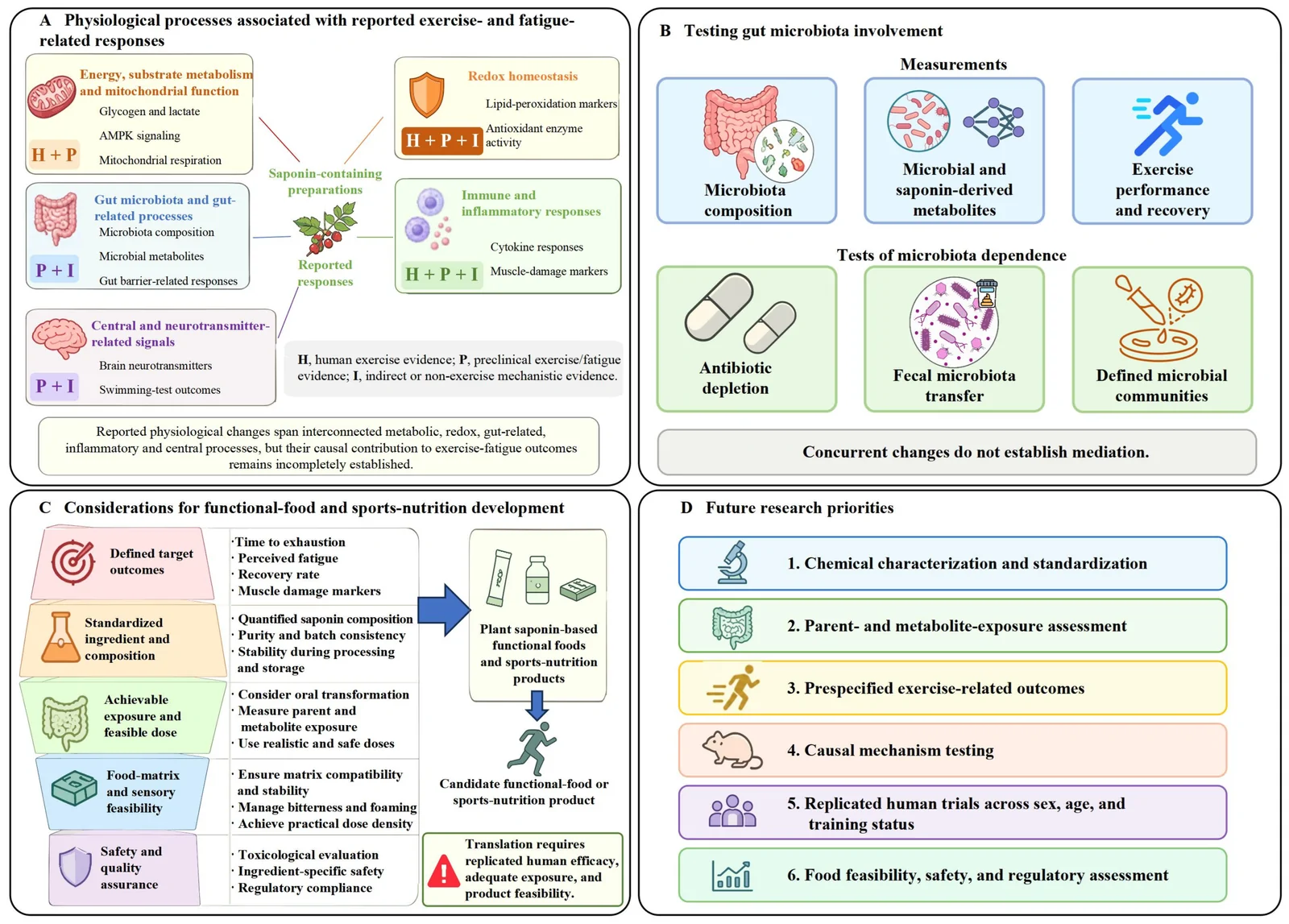
Reported physiological responses and research needs for plant saponins in exercise-related fatigue. (**A**) Measured responses and evidence sources; (**B**) measurements and experimental approaches for testing gut microbiota involvement; (**C**) considerations for functional-food and sports-nutrition products; (**D**) priorities for future research. H, human exercise studies; P, preclinical exercise or fatigue studies; I, indirect or non-exercise studies. The labels summarize evidence sources for each group of responses and do not indicate that every measure has been studied in humans or that causal mediation has been established.

Future studies could use antibiotic depletion, fecal microbiota transfer, gnotobiotic models, or defined microbial consortia to test whether a performance effect depends on the microbiota. Measurements of short-chain fatty acids, bile acids, tryptophan metabolites, and saponin-derived metabolites would help distinguish changes in microbial function from changes in saponin conversion and exposure (Figure 4B).

### 6.3. Redox Homeostasis and Mitochondrial Protection

In a long-term high-intensity swimming mouse model, gypenosides altered antioxidant-enzyme activity and lipid-peroxidation markers alongside longer exhaustive-swimming time [72]. A red-ginseng extract altered mitochondrial indices in a mouse model combining exercise, cold stress, and sleep deprivation [71], while ginsenoside Rg3 altered redox markers in aged rats with postoperative fatigue [79]. These two models provide indirect evidence because their fatigue responses cannot be attributed to exercise alone. Changes in redox or mitochondrial markers do not establish that they mediate an exercise-performance effect.

Showing that a saponin increases Nrf2 activity or downstream enzymes after exercise does not establish that Nrf2 mediates a performance effect. The same limitation applies to SIRT1, PGC-1α, and other mitochondrial regulatory pathways proposed from fatigue-related models. More convincing causal evidence would require temporal measurements together with pathway perturbation showing that modification of the pathway changes the exercise-related response.

Mitochondrial interpretation should combine molecular markers with functional measurements. Marker-based measures such as citrate synthase activity, mitochondrial DNA (mtDNA) copy number, and expression of biogenesis-related proteins are informative but should be complemented by direct assessments of mitochondrial function [32]. High-resolution respirometry, ATP production efficiency, membrane potential, mitochondrial ROS emission, and mitophagy flux would provide more direct assessment of mitochondrial function. In human studies, muscle biopsies are demanding, but peripheral blood metabolomics, near-infrared spectroscopy, 31P magnetic resonance spectroscopy, and indirect calorimetry could provide less invasive links among saponin intake, oxidative metabolism, and fatigue-related outcomes.

### 6.4. Immune and Inflammatory Responses

Exercise-related cytokine responses depend on workload, training status, and recovery; muscle-derived cytokines can also participate in metabolic communication [34,80]. In exercise models, *Platycodon* saponins reduced muscle damage and inflammatory responses after eccentric exercise [57], while gypenosides were associated with lower inflammatory markers [72]. These findings do not establish that immune or inflammatory changes mediate improved performance or recovery. Lower inflammatory markers may reflect reduced tissue damage rather than direct modulation of an inflammatory pathway.

Future studies should measure leukocyte redistribution, cytokine kinetics, mucosal immunity, and recovery across repeated exercise bouts. Sampling before exercise, immediately afterward, and during recovery is needed because transient cytokine elevations can be part of normal adaptation [80]. These measurements should be paired with recovery of muscle function.

### 6.5. Central and Neurotransmitter-Related Responses

In a long-term high-intensity swimming mouse model, gypenosides prolonged exhaustion time while decreasing serotonin (5-HT) and increasing dopamine (DA) in the frontal cortex [72]. A study using a mouse model described as chronic fatigue syndrome also reported longer exhaustive-swimming time and altered brain neurotransmitter profiles after ginseng stem and leaf saponin treatment [81]. These concurrent changes do not establish that the neurotransmitter responses mediate improved exercise performance or reduced central fatigue.

The physiological processes discussed in this section and the types of evidence supporting them are summarized in Figure 4A.

The mechanisms discussed in this section should not be treated as independent or equally supported explanations. Within the saponin literature reviewed here, evidence linking energy metabolism and mitochondrial function with exercise-related outcomes is comparatively more direct [46,52,53], whereas gut-microbiota evidence remains limited and largely associative [35,78]. Immune and central/neurotransmitter interpretations rely more heavily on associative or mechanistic evidence and have not been established as causal mediators of exercise-performance effects [72,81]. The main evidence and unresolved questions for each mechanism are summarized in Table 3.

## 7. Conclusions and Future Perspectives

Animal studies have reported changes in exercise duration and associated physiological measures [42,46,50,53,57,72]. Human evidence remains limited, with different results across exercise-related subjective, biochemical, and performance outcomes [45,51,52,54]. Small crossover trials of purified ginsenoside Rg1 provide preliminary evidence of acute endurance effects [68,69], whereas controlled *Tribulus terrestris* trials have not demonstrated consistent ergogenic benefits [60,61]. These findings do not establish an ergogenic effect shared by all dietary saponins. Small samples, limited replication, and incomplete chemical and exposure measurements constrain interpretation. The Rg1 trials predominantly involved young males. The applicability of the available findings to elite athletes remains uncertain.

Future studies should use chemically characterized preparations, quantify exposure to parent compounds and metabolites, and prespecify exercise-performance, perceived-fatigue, or recovery outcomes. Mechanistic work should test whether changes in the proposed pathway alter the exercise response. Human trials should recruit adequate numbers of women and older adults and include participants with different training backgrounds. Sex, age, and training status should be reported. Comparisons of exercise outcomes and adverse events across these groups should be planned in advance and supported by adequate sample sizes. Findings should also be replicated across relevant exercise settings. Food applications also require feasible doses, acceptable sensory properties, stability, and assessment of safety and regulatory status [13,23].

Safety findings apply to the specific preparation, dose, and duration tested. A four-week trial of *Panax ginseng* extract at 1 or 2 g/day reported mild events, including dyspepsia and insomnia, in both the ginseng and placebo groups [82]. A 24-week trial of Korean red ginseng at 2 g/day found no significant difference from placebo in the proportion of participants reporting adverse events (39.2% vs. 42.0%) or adverse drug reactions [83]. These results do not establish safety for other saponins, higher doses, longer use, or formulations that increase systemic exposure.

Adverse-event reporting also needs improvement: 23 of the 44 trials included in a systematic review did not mention adverse events [84]. Future exercise trials should record all adverse events, including gastrointestinal symptoms, sleep disturbance, headache, and skin reactions, together with their timing, severity, outcome, and possible relation to supplementation [82,83]. Reports should distinguish the number of affected participants from the total number of events and report serious events and withdrawals due to adverse events for each group.

Potential supplement–drug interactions require assessment of the specific preparation. In a randomized trial of 20 healthy volunteers, American ginseng (Panax quinquefolius) reduced plasma warfarin exposure and the anticoagulant response after two weeks [85]. This finding does not identify the responsible constituent or establish the same interaction for *Panax ginseng* or isolated saponins. Studies should record prescription medicines, non-prescription drugs, and other supplements at baseline and during follow-up. Use of ginseng-containing products by people taking warfarin should be reviewed with the prescribing clinician.

For athletic use, the product, dose, and timing should be reviewed with a sports physician or dietitian, and tolerability should be assessed during training before competition [7]. Selecting independently audited products can reduce the risk of exposure to undeclared prohibited substances, although it cannot eliminate that risk [7]. Such checks do not establish exercise-performance benefits. Food applications also require verified ingredient composition and quality assurance (Figure 4C), and regulatory requirements should be checked for the intended market [86].

The research priorities are summarized in Figure 4D. Negative findings are also informative. Failure to achieve the intended parent or metabolite exposure suggests an exposure-related limitation that may involve dose, formulation, absorption, adherence, or sampling. Conversely, when biologically relevant exposure is achieved in an adequately powered study but the prespecified primary exercise outcome does not improve, the result should not be reinterpreted as evidence of efficacy solely on the basis of unrelated antioxidant, anti-inflammatory, or other mechanistic biomarker changes.

## Figures and Tables

**Table 1:** Selected plant saponin sources: composition, oral exposure, and exercise-related evidence.

Plant Source or Material	Major Saponins and Processing	Oral Exposure	Exercise Evidence and Main Limitation	References
*Panax ginseng*	Mainly dammarane ginsenosides (PPD/PPT types); plant part, heating, and fermentation change the saponin profile.	Many parent ginsenosides are poorly absorbed and undergo intestinal or microbial deglycosylation.	Human studies report post-exercise subjective and biochemical changes; animal studies include performance tests. Whole-extract effects cannot be attributed to saponins alone.	[18,21,22,43,44,45,46]
*Gynostemma pentaphyllum*	Dammarane gypenosides; origin and heat processing alter the profile. Gypenoside L has been studied separately.	Rat studies demonstrate metabolism and low oral bioavailability for selected gypenosides; exposure in exercise trials is incompletely characterized.	Small human trials report performance-related effects; independent replication and attribution to individual gypenosides remain limited.	[37,38,47,48,49,50,51,52]
*Panax notoginseng*	Dammarane notoginsenosides and ginsenosides; processing changes composition.	Oral exposure varies with compound structure and biotransformation; exercise-specific exposure measurements remain limited.	Mouse studies report longer swimming endurance. A small human trial reported a within-group endurance increase; this comparison does not establish superiority to placebo.	[19,53,54]
*Platycodon grandiflorus*	Oleanane-type platycodins; extraction and purification determine preparation composition, while bitterness may constrain food use.	Platycodin D has low oral bioavailability in rats (~1.9%); exercise-specific exposure data remain lacking.	Two mouse studies reported reduced eccentric-exercise muscle damage or improved strength/endurance. Human efficacy remains untested in the reviewed literature.	[13,40,55,56,57,58]
*Tribulus terrestris*	Steroidal saponins, mainly spirostanol/furostanol types; composition varies among origins and commercial preparations.	Individual saponin exposure is poorly characterized in the exercise studies.	Improved endurance was reported in overtrained rats, but controlled human studies found no consistent performance benefit.	[41,42,59,60,61]
*Quillaja saponins*	Triterpenoid saponins used for foaming and emulsification; purification and composition affect technological properties.	Not assessed in exercise studies reviewed here.	Included as a food-functionality comparator; no exercise-fatigue benefit is established.	[12,13]

PPD, protopanaxadiol; PPT, protopanaxatriol.

**Table 2:** Representative human and animal studies of saponin-containing preparations and purified compounds in exercise performance, fatigue, and muscle damage.

Population/Model and Design	Preparation and Regimen	Exercise Context	Main Finding	Main Limitation	References
Healthy Korean adults; 100 randomized (88 men, 12 women), 93 protocol completers; double-blind parallel RCT	*Gynostemma pentaphyllum* extract, 450 mg/day for 12 weeks; gypenoside L 18.46 mg/g and Rg3 1.61 mg/g	Treadmill cardiopulmonary testing	VO_2_max and oxygen pulse were higher than placebo (*p* = 0.007 and *p* = 0.047).	Sex imbalance and endpoint-specific exclusions; effects cannot be assigned to individual saponins.	[51]
Untrained young men; 19 enrolled, 16 completed; double-blind crossover RCT; 4-week washout	*G. pentaphyllum* leaf extract, 450 mg/day for 4 weeks; 20(S)-Rg3 0.24% and gypenosides 2.14%	20 km cycling time trial; cycling to exhaustion	Time-trial completion: 2634 ± 101 versus 2747 ± 121 s (approximately 4% faster; *p* < 0.05). Time to exhaustion did not differ significantly.	Small male sample; changes in muscle metabolism do not establish the cause of improved performance.	[52]
Adults; 110 randomized, 107 analyzed (41 men, 66 women); mean age above 50 years; double-blind parallel RCT	Korean red ginseng, 3 capsules (465 mg/capsule) twice daily for 8 weeks; total saponins 3.5–4.8%	Graded cycling challenge	After 8 weeks, the post-exercise physical-perception score was lower (10.46 ± 0.61 versus 10.92 ± 0.47; *p* < 0.01), indicating less perceived fatigue; lactate and CK were also lower (*p* < 0.05).	Subjective and biochemical outcomes do not establish improved exercise performance.	[45]
Untrained young men; *n* = 12 for performance; double-blind crossover RCT; 4-week washout	Purified Rg1, 5 mg the night before and 5 mg 1 h before exercise	Cycling to exhaustion at 80% VO_2_max	Time to exhaustion: 38.3 ± 6.7 versus 31.8 ± 5.0 min (*p* = 0.04); VO_2_max did not differ significantly.	Small male sample; acute dosing; causal role of molecular changes in the performance effect remains unclear.	[68]
Young men; separate performance and biopsy cohorts, *n* = 12 each; double-blind crossover RCT; ≥4-week washout	Purified Rg1, 5 mg 1 h before exercise	Cycling to exhaustion at 80% VO_2_max	Time to exhaustion increased by 12% (*p* < 0.05).	Small male samples; acute dosing; separate cohorts prevent direct linkage of biopsy findings to performance.	[69]
Untrained adults; *n* = 29 (13 intervention, 16 placebo); double-blind parallel RCT	*Panax notoginseng* preparation, 1350 mg/day for 30 days	Cycling endurance test	Endurance time increased by >7 min from baseline in the intervention group (*p* < 0.05).	Within-group change does not establish an advantage over placebo; small sample.	[54]
Resistance-trained men; *n* = 15; parallel RCT	*Tribulus terrestris* preparation, 3.21 mg/kg/day for 8 weeks	Periodized resistance training	No advantage over placebo in exercise performance or body composition.	Small male sample; findings apply to the tested preparation and training regimen.	[60]
University athletes; *n* = 13; double-blind crossover RCT; 2-week washout	*T. terrestris* preparation, 20 mg/kg three times daily for 4 weeks	Performance testing after a detraining period	No improvement in VO_2_max, time to exhaustion, muscle strength, or body composition.	Small sample; short intervention; findings apply to the tested preparation and regimen.	[61]
Male Kunming mice; *n* = 8/group per endpoint; vehicle control	*P. notoginseng* total saponins, 20, 40, or 80 mg/kg/day by gavage for 28 days	Exhaustive swimming	Swimming time increased by 21%, 27%, and 34%; increases at 40 and 80 mg/kg were significant (*p* < 0.05).	Forced-swimming findings do not establish effects at comparable human exposure.	[53]
Male ICR mice; *n* = 8/group; vehicle control	Changbai Mountain ginseng extract, 5 or 25 mg/kg/day orally for 4 weeks	Swimming endurance test	Swimming time was 4.1 and 4.0 min versus 2.5 min in controls (both *p* < 0.05).	The contribution of saponins cannot be separated from other extract constituents.	[46]
Male ICR mice; *n* = 10/group; exercise plus vehicle control	*G. pentaphyllum* extract, 300 mg/kg/day, or purified gypenoside L, 7 mg/kg/day, by gavage for 6 weeks	Treadmill endurance test	Endurance time was 1.46-fold with the extract and 1.22-fold with gypenoside L relative to control.	Unmatched exposure prevents a comparison of intrinsic potency.	[50]
ICR mice; *n* = 8/group; exercise plus vehicle control	Gypenosides (≥95% purity), 100 mg/kg/day by gavage for 4 weeks	Repeated swimming; exhaustive-swimming test	Swimming time was longer (*p* < 0.01), with higher liver and muscle glycogen.	Multiple associated pathways were measured; their contribution to performance was not established.	[72]
Male Sprague–Dawley rats; *n* = 10/group; overtraining plus saline control	*T. terrestris* extract (>70% saponins), 120 mg/kg before each training session for 6 weeks	Progressive overtraining; treadmill exhaustion test	Time to exhaustion was significantly longer than in the overtraining control group.	Single rat model; findings do not establish efficacy at human dietary exposure.	[42]
Male ICR mice; exercise plus PBS control and resting control	*Platycodon* saponins, 4 mg/kg; 2-week supplementation	Eccentric exercise-induced muscle damage	Muscle-damage area and serum LDH, CK, and CRP were lower than in exercise controls.	Histological and biochemical findings do not establish improved functional recovery or endurance.	[57]
Mice; *n* = 7/group; vehicle control	Purified 20(S)-PPD or 20(S)-PPT, 5 or 10 mg/kg/day orally for 5 days	Weight-loaded swimming and rotarod tests	PPT prolonged swimming time, whereas PPD did not.	Short study without matched exposure; results do not establish a general PPD/PPT potency ranking.	[73]
Male Kunming mice; reported total *n* = 40; vehicle control	Purified 20(S)-Rg3, 10, 20, or 40 mg/kg/day by gavage for 28 days	Forced-swimming test	Swimming time was 203.6–248.8 min across doses versus 168.4 min in controls (all *p* < 0.05).	Per-group sample size was inconsistently reported (8 versus 10); no direct stereoisomer comparison or confirmation at food-achievable exposure.	[74]

Human studies used placebo comparators. Findings are between-treatment comparisons unless a within-group change is specified. Values are mean ± SEM for [52,68] and mean ± SD for [45]; animal performance values are summarized without dispersion. SD, standard deviation; SEM, standard error of the mean; RCT, randomized controlled trial; VO_2_max, maximal oxygen uptake; CK, creatine kinase; LDH, lactate dehydrogenase; CRP, C-reactive protein; PBS, phosphate-buffered saline; PPD, protopanaxadiol; PPT, protopanaxatriol. Botanical preparations should not be treated as equivalent to isolated saponins, and animal doses should not be interpreted as human dietary doses. Studies [68,69] received industry-linked funding; Ref. [69] also disclosed patent-related interests.

**Table 3:** Exercise-related findings, limitations, and research needs for proposed physiological effects of saponin-containing interventions.

Physiological Process	Exercise-Related Findings	Main Limitation	Next Measurements or Tests	References
Energy and substrate metabolism	*Panax notoginseng* total saponins and ginseng extracts altered glycogen and lactate-related measures in exercising mice. In rats undergoing repeated loaded swimming, *Panax ginseng* root extract altered glycogen availability and PI3K/Akt/mTOR-related proteins.	Post-exercise glycogen and lactate depend on work performed and sampling time; these measures do not identify substrate flux.	Compare matched workloads; measure substrate oxidation, lactate turnover, and glycogen before exercise and during recovery.	[4,27,46,53,70]
AMPK signaling and mitochondrial respiration	In untrained men, *Gynostemma pentaphyllum* leaf extract improved 20 km cycling time-trial performance. Oxygen flux was higher in muscle sampled at rest; AMPK Thr172 phosphorylation increased after 60 min of exercise relative to pre-exercise values during extract supplementation.	Whole-extract effects cannot be assigned to individual saponins; the contribution of AMPK or respiratory changes to performance was not tested.	Combine repeated muscle sampling with respirometry and ATP-production measurements; test pathway dependence alongside exercise outcomes.	[27,32,52]
Gut microbiota and intestinal responses	In rats undergoing loaded swimming, ginseng water extract changed gut microbiota and fatigue-associated biochemical and metabolic measures.	The reported microbiota and metabolic changes do not establish a causal link to exercise performance or identify saponins as the active constituents.	Measure exercise performance, microbial metabolites, and intestinal permeability; test microbiota dependence while quantifying saponin exposure.	[35,78]
Redox regulation	Rg1 attenuated the post-exercise rise in a muscle lipid-peroxidation marker in young men. Gypenosides altered antioxidant-enzyme activity and lipid-peroxidation markers alongside longer swimming time in mice.	Human evidence is from a small acute trial; marker changes do not establish improved redox control or explain the performance effect.	Compare matched workloads and recovery times; combine redox measures with pathway perturbation and functional outcomes.	[29,30,31,68,72]
Immune and inflammatory responses	Rg1 altered post-exercise muscle TNF-α and IL-10 mRNA responses in young men. *Platycodon* saponins reduced muscle damage after eccentric exercise in mice; gypenosides reduced inflammatory markers in a swimming model.	Lower inflammatory markers may reflect reduced tissue damage; mediation of performance or functional recovery remains untested.	Measure cytokines, leukocyte responses, muscle damage, and recovery of force at matched time points.	[57,68,72,77,80]
Central and neurotransmitter-related responses	Gypenosides and ginseng stem/leaf saponins were associated with longer exhaustive-swimming time and altered brain neurotransmitters in mice; the latter study used a model described as chronic fatigue syndrome.	Neurotransmitter concentrations do not establish reduced central fatigue; differences between fatigue models limit comparison.	Pair neurochemical time courses with standardized exercise tests; include perceived fatigue and voluntary activation in human studies.	[2,36,72,81]

Associated changes do not establish causal mediation. AMPK, AMP-activated protein kinase; ATP, adenosine triphosphate; PI3K, phosphoinositide 3-kinase; Akt, protein kinase B; mTOR, mechanistic target of rapamycin; TNF-α, tumor necrosis factor α; IL-10, interleukin 10.

## Data Availability

No new data were created or analyzed in this study. Data sharing is not applicable to this article.

## References

[1] Hargreaves M., Spriet L.L. (2018). Exercise metabolism: Fuels for the fire. Cold Spring Harb. Perspect. Med..

[2] Enoka R.M., Duchateau J. (2016). Translating fatigue to human performance. Med. Sci. Sports Exerc..

[3] Tornero-Aguilera J.F., Jimenez-Morcillo J., Rubio-Zarapuz A., Clemente-Suárez V.J. (2022). Central and peripheral fatigue in physical exercise explained: A narrative review. Int. J. Environ. Res. Public Health.

[4] Allen D.G., Lamb G.D., Westerblad H. (2008). Skeletal muscle fatigue: Cellular mechanisms. Physiol. Rev..

[5] Gomez-Cabrera M.C., Carretero A., Millan-Domingo F., Garcia-Dominguez E., Correas A.G., Olaso-Gonzalez G., Viña J. (2021). Redox-related biomarkers in physical exercise. Redox Biol..

[6] Yan K., Gao H., Liu X., Zhao Z., Gao B., Zhang L. (2022). Establishment and identification of an animal model of long-term exercise-induced fatigue. Front. Endocrinol..

[7] Maughan R.J., Burke L.M., Dvorak J., Larson-Meyer D.E., Peeling P., Phillips S.M., Rawson E.S., Walsh N.P., Garthe I., Geyer H. (2018). IOC consensus statement: Dietary supplements and the high-performance athlete. Br. J. Sports Med..

[8] Guest N.S., VanDusseldorp T.A., Nelson M.T., Grgic J., Schoenfeld B.J., Jenkins N.D.M., Arent S.M., Antonio J., Stout J.R., Trexler E.T. (2021). International society of sports nutrition position stand: Caffeine and exercise performance. J. Int. Soc. Sports Nutr..

[9] Kreider R.B., Kalman D.S., Antonio J., Ziegenfuss T.N., Wildman R., Collins R., Candow D.G., Kleiner S.M., Almada A.L., Lopez H.L. (2017). International Society of Sports Nutrition position stand: Safety and efficacy of creatine supplementation in exercise, sport, and medicine. J. Int. Soc. Sports Nutr..

[10] Trexler E.T., Smith-Ryan A.E., Stout J.R., Hoffman J.R., Wilborn C.D., Sale C., Kreider R.B., Jäger R., Earnest C.P., Bannock L. (2015). International society of sports nutrition position stand: Beta-alanine. J. Int. Soc. Sports Nutr..

[11] Sellami M., Slimeni O., Pokrywka A., Kuvačić G., Hayes L.D., Milic M., Padulo J. (2018). Herbal medicine for sports: A review. J. Int. Soc. Sports Nutr..

[12] Reichert C.L., Salminen H., Weiss J. (2019). *Quillaja* saponin characteristics and functional properties. Annu. Rev. Food Sci. Technol..

[13] Timilsena Y.P., Phosanam A., Stockmann R. (2023). Perspectives on saponins: Food functionality and applications. Int. J. Mol. Sci..

[14] Wang J., Tang X., Liu F., Mao B., Zhang Q., Zhao J., Chen W., Cui S. (2024). Sources, metabolism, health benefits and future development of saponins from plants. Food Res. Int..

[15] You Q.-Y., Qi Y.-H., Wu J., Zou L., Wang Z.-P., Wan Y., Huang J.-W. (2026). Saponins: Advanced extraction, quantification techniques, multi-target bioactivities, and applications in food and health. Food Chem..

[16] Jin T.-Y., Rong P.-Q., Liang H.-Y., Zhang P.-P., Zheng G.-Q., Lin Y. (2020). Clinical and preclinical systematic review of *Panax ginseng* C.A. Mey and its compounds for fatigue. Front. Pharmacol..

[17] Zhang Z.-B., Yu C.-Y., Wang H.-Y., Jia X.-B., Wu W., Pang S.-T., Li W., Zahoor S., Khan W., Liu Y.-C. (2025). The history, beneficial ingredients, mechanism, processing, and products of *Panax ginseng* for medicinal and edible value. Food Med. Homol..

[18] Piao X., Zhang H., Kang J.P., Yang D.U., Li Y., Pang S., Jin Y., Yang D.C., Wang Y. (2020). Advances in saponin diversity of *Panax ginseng*. Molecules.

[19] Peng M., Yi Y.X., Zhang T., Ding Y., Le J. (2018). Stereoisomers of saponins in *Panax notoginseng* (Sanqi): A review. Front. Pharmacol..

[20] Geng X., Wang J., Liu Y., Liu L., Liu X., Zhao Y., Wang C., Liu J. (2024). Research progress on chemical diversity of saponins in *Panax ginseng*. Chin. Herb. Med..

[21] Hyun S.H., Kim S.W., Seo H.W., Youn S.H., Kyung J.S., Lee Y.Y., In G., Park C.-K., Han C.-K. (2020). Physiological and pharmacological features of the non-saponin components in Korean Red Ginseng. J. Ginseng Res..

[22] He Y., Hu Z., Li A., Zhu Z., Yang N., Ying Z., He J., Wang C., Yin S., Cheng S. (2019). Recent advances in biotransformation of saponins. Molecules.

[23] Mao T.-L., Li J., Guo X., Wu Q.-L., Thakur K., Ma Y.-L., Wei Z.-J. (2026). Recent advances in dietary saponins stabilization: A review on technologies and applications. Trends Food Sci. Technol..

[24] Behrens M., Gube M., Chaabene H., Prieske O., Zenon A., Broscheid K.-C., Schega L., Husmann F., Weippert M. (2023). Fatigue and human performance: An updated framework. Sports Med..

[25] Poole D.C., Copp S.W., Colburn T.D., Craig J.C., Allen D.L., Sturek M., O’Leary D.S., Zucker I.H., Musch T.I. (2020). Guidelines for animal exercise and training protocols for cardiovascular studies. Am. J. Physiol.-Heart Circ. Physiol..

[26] Kellmann M., Bertollo M., Bosquet L., Brink M., Coutts A.J., Duffield R., Erlacher D., Halson S.L., Hecksteden A., Heidari J. (2018). Recovery and performance in sport: Consensus statement. Int. J. Sports Physiol. Perform..

[27] Hargreaves M., Spriet L.L. (2020). Skeletal muscle energy metabolism during exercise. Nat. Metab..

[28] Sundberg C.W., Fitts R.H. (2019). Bioenergetic basis of skeletal muscle fatigue. Curr. Opin. Physiol..

[29] Margaritelis N.V., Paschalis V., Theodorou A.A., Kyparos A., Nikolaidis M.G. (2020). Redox basis of exercise physiology. Redox Biol..

[30] Meng Q., Su C.-H. (2024). The impact of physical exercise on oxidative and nitrosative stress: Balancing the benefits and risks. Antioxidants.

[31] Gallego-Selles A., Martin-Rincon M., Martinez-Canton M., Perez-Valera M., Martín-Rodríguez S., Gelabert-Rebato M., Santana A., Morales-Alamo D., Dorado C., Calbet J.A.L. (2020). Regulation of Nrf2/Keap1 signalling in human skeletal muscle during exercise to exhaustion in normoxia, severe acute hypoxia and post-exercise ischaemia: Influence of metabolite accumulation and oxygenation. Redox Biol..

[32] Philp A.M., Saner N.J., Lazarou M., Ganley I.G., Philp A. (2021). The influence of aerobic exercise on mitochondrial quality control in skeletal muscle. J. Physiol..

[33] Yu M., Jiang X., Zhang Y., Zhang W., Wang T., Wang J., Shao J., Zhang L., Sun Y., Meng X. (2025). Mitophagy as a therapeutic target for exercise-induced fatigue: Modulation by natural compounds and mechanistic insights. Front. Physiol..

[34] Severinsen M.C.K., Pedersen B.K. (2020). Muscle–organ crosstalk: The emerging roles of myokines. Endocr. Rev..

[35] Zhao Z., Zhao S., Li W., Lai Z., Zhou Y., Guan F., Liang X., Zhang J., Wang L. (2026). Gut microbiota and exercise-induced fatigue: A narrative review of mechanisms, nutritional interventions, and future directions. Nutrients.

[36] Martin K.S., Azzolini M., Lira Ruas J. (2020). The kynurenine connection: How exercise shifts muscle tryptophan metabolism and affects energy homeostasis, the immune system, and the brain. Am. J. Physiol.-Cell Physiol..

[37] Su C., Li N., Ren R., Wang Y., Su X., Lu F., Zong R., Yang L., Ma X. (2021). Progress in the medicinal value, bioactive compounds, and pharmacological activities of *Gynostemma pentaphyllum*. Molecules.

[38] Chen X.-B., Yao C.-L., Hou J.-R., Nie M., Li Y., Wei W.-L., Zhang J.-Q., Qu H., Li J.-Y., Bi Q.-R. (2023). Systematical characterization of gypenosides in *Gynostemma pentaphyllum* and the chemical composition variation of different origins. J. Pharm. Biomed. Anal..

[39] Liang H.-Z., Wang M.-Y., Yang G., Li G., Zhang J., Yao L., Nan Y., Huang C.-L., Chen X.-J., Song J. (2025). Untargeted qualitative and targeted quantitative analysis of saponins reveal differential chemotypes of *Gynostemma pentaphyllum* and *G. longipes* from different geographical origins. Food Chem..

[40] Ji M.-Y., Bo A., Yang M., Xu J.-F., Jiang L.-L., Zhou B.-C., Li M.-H. (2020). The pharmacological effects and health benefits of *Platycodon grandiflorus*—A medicine food homology species. Foods.

[41] Dinchev D., Janda B., Evstatieva L., Oleszek W., Aslani M.R., Kostova I. (2008). Distribution of steroidal saponins in *Tribulus terrestris* from different geographical regions. Phytochemistry.

[42] Yin L., Wang Q., Wang X., Song L.-N. (2016). Effects of *Tribulus terrestris* saponins on exercise performance in overtraining rats and the underlying mechanisms. Can. J. Physiol. Pharmacol..

[43] Xu X.-F., Gao Y., Xu S.-Y., Liu H., Xue X., Zhang Y., Zhang H., Liu M.-N., Xiong H., Lin R.-C. (2018). Remarkable impact of steam temperature on ginsenosides transformation from fresh ginseng to red ginseng. J. Ginseng Res..

[44] Kim D.-H. (2018). Gut microbiota-mediated pharmacokinetics of ginseng saponins. J. Ginseng Res..

[45] Yang Y., Wang H., Zhang M., Shi M., Yang C., Ni Q., Wang Q., Li J., Wang X., Zhang C. (2022). Safety and antifatigue effect of Korean Red Ginseng capsule: A randomized, double-blind and placebo-controlled clinical trial. J. Ginseng Res..

[46] Ma G.-D., Chiu C.-H., Hsu Y.-J., Hou C.-W., Chen Y.-M., Huang C.-C. (2017). Changbai Mountain Ginseng (*Panax ginseng* C.A. Mey) extract supplementation improves exercise performance and energy utilization and decreases fatigue-associated parameters in mice. Molecules.

[47] Duan Y., Yang J., Xie J.-B., Xie P., Qi Y.-S., Zhao M.-T., Piao X.-L. (2021). Simultaneous quantitative analysis of nine saponins in *Gynostemma pentaphyllum* before and after heat processing based on UPLC-Q-Trap-MS. Zhongguo Zhong Yao ZA Zhi.

[48] Chen D.-J., Hu H.-G., Xing S.-F., Gao Y.-J., Xu S.-F., Piao X.-L. (2014). Metabolic profiling of *Gynostemma pentaphyllum* extract in rat serum, urine and faeces after oral administration. J. Chromatogr. B.

[49] He Y., Liang Q., Luo L., He Y., Huang X., Wen C. (2022). Determination of Gypenoside A and Gypenoside XLIX in rat plasma by UPLC-MS/MS and applied to the pharmacokinetics and bioavailability. Int. J. Anal. Chem..

[50] Kim Y.H., Jung J.I., Jeon Y.E., Kim S.M., Hong S.H., Kim T.Y., Kim E.J. (2022). *Gynostemma pentaphyllum* extract and its active component gypenoside L improve the exercise performance of treadmill-trained mice. Nutr. Res. Pract..

[51] Ahn Y., Lee H.-S., Lee S.-H., Joa K.-L., Lim C.Y., Ahn Y.J., Suh H.J., Park S.-S., Hong K.-B. (2023). Effects of gypenoside L-containing *Gynostemma pentaphyllum* extract on fatigue and physical performance: A double-blind, placebo-controlled, randomized trial. Phytother. Res..

[52] Nayyar D., Yan X., Xu G., Shi M., Garnham A.P., Mathai M.L., McAinch A.J. (2023). *Gynostemma pentaphyllum* increases exercise performance and alters mitochondrial respiration and AMPK in healthy males. Nutrients.

[53] Xu Y.-X., Zhang J.-J. (2013). Evaluation of anti-fatigue activity of total saponins of Radix notoginseng. Indian J. Med. Res..

[54] Liang M.T.C., Podolka T.D., Chuang W.J. (2005). *Panax notoginseng* supplementation enhances physical performance during endurance exercise. J. Strength Cond. Res..

[55] Cheok C.Y., Salman H.A.K., Sulaiman R. (2014). Extraction and quantification of saponins: A review. Food Res. Int..

[56] Kwon M., Ji H.-K., Goo S.H., Nam S.J., Kang Y.J., Lee E., Liu K.H., Choi M.-K., Song I.-S. (2017). Involvement of intestinal efflux and metabolic instability in the pharmacokinetics of platycodin D in rats. Drug Metab. Pharmacokinet..

[57] Kim Y.-A., Oh S.-H., Lee G.-H., Hoa P.T., Jin S.W., Chung Y.C., Lee Y.C., Jeong H.-G. (2018). *Platycodon* grandiflorum-derived saponin attenuates the eccentric exercise-induced muscle damage. Food Chem. Toxicol..

[58] Kim Y.-A., Jin S.W., Oh S.-H., Lee G.-H., Pham H.T., Choi J.H., Chung Y.C., Lee W.L., Kim S.K., Jeong H.-G. (2018). *Platycodon* grandiflorum-derived saponin enhances exercise function, skeletal muscle protein synthesis, and mitochondrial function. Food Chem. Toxicol..

[59] Ganzera M., Bedir E., Khan I.A. (2001). Determination of steroidal saponins in *Tribulus terrestris* by reversed-phase high-performance liquid chromatography and evaporative light scattering detection. J. Pharm. Sci..

[60] Antonio J., Uelmen J., Rodriguez R., Earnest C.P. (2000). The effects of *Tribulus terrestris* on body composition and exercise performance in resistance-trained males. Int. J. Sport Nutr. Exerc. Metab..

[61] Ataei L., Giannaki C.D., Petrou C., Aphamis G. (2025). *Tribulus terrestris* failed to improve exercise performance and body composition following a detraining period. Sport Sci. Health.

[62] Heinrich M., Jalil B., Abdel-Tawab M., Echeverria J., Kulić Ž., McGaw L.J., Pezzuto J.M., Potterat O., Wang J.-B. (2022). Best practice in the chemical characterisation of extracts used in pharmacological and toxicological research—The ConPhyMP—Guidelines. Front. Pharmacol..

[63] Wei W., Li M., Pan L., Shao M., He X., Li Y., Sheng L., Zheng N., Li H. (2025). Inter-species and individualized biotransformation of five saponins by human being- and mouse-derived fecal microbiota. Chin. Med..

[64] Capuano E., Janssen A.E.M. (2021). Food matrix and macronutrient digestion. Annu. Rev. Food Sci. Technol..

[65] Carter S., Solomon T.P.J. (2019). In vitro experimental models for examining the skeletal muscle cell biology of exercise: The possibilities, challenges and future developments. Pflüg. Arch.—Eur. J. Physiol..

[66] Lee Y.-G., Hong W., Cho Y.M., Kwon J.E., Yang D.-C., Kim H., Kang S.C. (2025). Anti-fatigue effects of Korean Red Ginseng extract in healthy Japanese adults: A randomized, double-blind, placebo-controlled study. J. Ginseng Res..

[67] Zhang L., Chen X., Cheng Y., Chen Q., Tan H., Son D., Chang D., Bian Z., Fang H., Xu H. (2019). Safety and antifatigue effect of Korean Red Ginseng: A randomized, double-blind, and placebo-controlled clinical trial. J. Ginseng Res..

[68] Hou C.-W., Lee S.-D., Kao C.-L., Cheng I.-S., Lin Y.-N., Chuang S.-J., Chen C.-Y., Ivy J.L., Huang C.-Y., Kuo C.-H. (2015). Improved inflammatory balance of human skeletal muscle during exercise after supplementations of the ginseng-based steroid Rg1. PLoS ONE.

[69] Wu J., Saovieng S., Cheng I.-S., Liu T., Hong S., Lin C.-Y., Su I.-C., Huang C.-Y., Kuo C.-H. (2019). Ginsenoside Rg1 supplementation clears senescence-associated β-galactosidase in exercising human skeletal muscle. J. Ginseng Res..

[70] Zhang G., Lu B., Wang E., Wang W., Li Z., Jiao L., Li H., Wu W. (2023). *Panax ginseng* improves physical recovery and energy utilization on chronic fatigue in rats through the PI3K/AKT/mTOR signalling pathway. Pharm. Biol..

[71] Zhang H., Zhao C., Hou J., Su P., Yang Y., Xia B., Zhao X., He R., Wang L., Cao C. (2022). Red ginseng extract improves skeletal muscle energy metabolism and mitochondrial function in chronic fatigue mice. Front. Pharmacol..

[72] Li Y., Ren Y., Liu R., Xu W., Gong Y. (2025). Alleviation of gypenosides on peripheral and central fatigue via anti-inflammation, anti-oxidation and neurotransmitter regulation. Food Sci. Nutr..

[73] Oh H.A., Kim D.-E., Choi H.J., Kim N.J., Kim D.-H. (2015). Anti-fatigue effects of 20(S)-protopanaxadiol and 20(S)-protopanaxatriol in mice. Biol. Pharm. Bull..

[74] Li S., Chen Z. (2018). Evaluation of antifatigue effects of 20(S)-ginsenoside Rg3 in forced swimming mice. Indian J. Pharm. Sci..

[75] Kim H.J., Oh T.K., Kim Y.H., Lee J., Moon J.M., Park Y.S., Sung C.M. (2022). Pharmacokinetics of ginsenoside Rb1, Rg3, Rk1, Rg5, F2, and compound K from red ginseng extract in healthy Korean volunteers. Evid.-Based Complement. Altern. Med..

[76] Tang W., Zhang Y., Gao J., Ding X., Gao S. (2008). The anti-fatigue effect of 20(R)-ginsenoside Rg3 in mice by intranasally administration. Biol. Pharm. Bull..

[77] Peake J.M., Neubauer O., Della Gatta P.A., Nosaka K. (2017). Muscle damage and inflammation during recovery from exercise. J. Appl. Physiol..

[78] Zhou S.-S., Zhou J., Xu J.-D., Shen H., Kong M., Yip K.-M., Han Q.-B., Zhao Z.-Z., Xu J., Chen H.-B. (2021). Ginseng ameliorates exercise-induced fatigue potentially by regulating the gut microbiota. Food Funct..

[79] Yang Q.-Y., Lai X.-D., Ouyang J., Yang J.-D. (2018). Effects of ginsenoside Rg3 on fatigue resistance and SIRT1 in aged rats. Toxicology.

[80] Phelps C.M., Meisel M. (2026). The immunology of exercise: Mechanisms, mediators, and therapeutic opportunities. Immunity.

[81] Zhang M., Liu R., Zhou T., Guo Y., Li H., Wu W. (2025). Exploring the anti-fatigue properties of ginseng stem and leaf saponins: Using UHPLC-MS metabolomics and neurotransmitter analysis. Phytomedicine.

[82] Lee N.-H., Yoo S.-R., Kim H.-G., Cho J.-H., Son C.-G. (2012). Safety and tolerability of *Panax ginseng* root extract: A randomized, placebo-controlled, clinical trial in healthy Korean volunteers. J. Altern. Complement. Med..

[83] Song S.-W., Kim H.-N., Shim J.-Y., Yoo B.-Y., Kim D.-H., Lee S.-H., Park J.-S., Kim M.-J., Yoo J.-H., Cho B. (2018). Safety and tolerability of Korean Red Ginseng in healthy adults: A multicenter, double-blind, randomized, placebo-controlled trial. J. Ginseng Res..

[84] Kim Y.-S., Woo J.-Y., Han C.-K., Chang I.-M. (2015). Safety analysis of *Panax ginseng* in randomized clinical trials: A systematic review. Medicines.

[85] Yuan C.-S., Wei G., Dey L., Karrison T., Nahlik L., Maleckar S., Kasza K., Ang-Lee M., Moss J. (2004). Brief communication: American ginseng reduces warfarin’s effect in healthy patients: A randomized, controlled trial. Ann. Intern. Med..

[86] Sun X.-Y., Xiang X.-S., Zhou Y.-J., Wen J.-Y., Sun G.-J. (2026). Global policy changes on homologous substances of food and medicine. Food Med. Homol..

